# Complete genome sequence of the *Sulfodiicoccus acidiphilus* strain HS-1^T^, the first crenarchaeon that lacks *polB3*, isolated from an acidic hot spring in Ohwaku-dani, Hakone, Japan

**DOI:** 10.1186/s13104-019-4488-5

**Published:** 2019-07-22

**Authors:** Hiroyuki D. Sakai, Norio Kurosawa

**Affiliations:** 1 Department of Science and Engineering for Sustainable Innovation, Faculty of Science and Engineering, Soka University, 1-236 Tangi-machi, Hachioji, Tokyo 192-8577 Japan; 2 Japan Society for the Promotion of Science, Chiyoda-ku, Tokyo, 102-8471 Japan

**Keywords:** *Crenarchaeota*, *Sulfolobales*, *Sulfodiicoccus*, *polB3*, *cdc6*, 3-Hydroxypropionate/4-hydroxybutyrate, Dicarboxylate/4-hydroxybutyrate

## Abstract

**Objective:**

*Sulfodiicoccus acidiphilus* HS-1^T^ is the type species of the genus *Sulfodiicoccus*, a thermoacidophilic archaeon belonging to the order *Sulfolobales* (class *Thermoprotei*; phylum *Crenarchaeota*). While *S. acidiphilus* HS-1^T^ shares many common physiological and phenotypic features with other *Sulfolobales* species, the similarities in their 16S rRNA gene sequences are less than 89%. In order to know the genomic features of *S. acidiphilus* HS-1^T^ in the order *Sulfolobales*, we determined and characterized the genome of this strain.

**Results:**

The circular genome of *S. acidiphilus* HS-1^T^ is comprised of 2353,189 bp with a G+C content of 51.15 mol%. A total of 2459 genes were predicted, including 2411 protein coding and 48 RNA genes. The notable genomic features of *S. acidiphilus* HS-1^T^ in *Sulfolobales* species are the absence of genes for polB3 and the autotrophic carbon fixation pathway, and the distribution pattern of essential genes and sequences related to genomic replication initiation. These insights contribute to an understanding of archaeal genomic diversity and evolution.

**Electronic supplementary material:**

The online version of this article (10.1186/s13104-019-4488-5) contains supplementary material, which is available to authorized users.

## Introduction

*Sulfodiicoccus acidiphilus* HS-1^T^, represented a novel genus, was recently isolated in our laboratory and validly described [1]. The genus belongs to the order *Sulfolobales*, a well-known taxon of the phylum *Crenarchaeota*, widely inhabits hot acidic environments all over the world [2–5]. The 16S rRNA gene sequence similarities between *S. acidiphilus* and other species in the order *Sulfolobales* were less than 89%. Given the low 16S rRNA gene similarities with other *Sulfolobales* species, we hypothesized that the strain also harbored distinct genomic features in *Sulfolobales* species. Therefore, we determined the complete genome of *S. acidiphilus* HS-1^T^ and compared it with other genomes in *Sulfolobales* species. Genomic analysis revealed that *S. acidiphilus* HS-1^T^ has several distinguishing genomic features.

## Main text

### Methods

#### Organism information

The isolation and characterization of *S. acidophilus* HS-1^T^ representing a novel genus *Sulfodiicoccus* was reported previously [1, 6]. The phylogenetic position of *S. acidiphilus* based on 16S rRNA gene sequences is shown in Additional file 1: Figure S1. The general features of *S. acidiphilus* HS-1^T^ are shown in Additional file 1: Table S1. HS-1^T^ is an irregular cocci, non-motile, thermoacidophilic archaeon. Optimal growth occurs at 65–70 °C and pH 3–3.5. The strain is obligately aerobic and can utilize the following organics as a sole carbon source: yeast extract, beef extract, casamino acids, peptone, tryptone, xylose, galactose, glucose, maltose, sucrose, raffinose, lactose, aspartic acid and glutamic acid. Chemolithotrophic growth does not occur when S^0^, FeS_2_, K_2_S_4_O_6_, Na_2_S_2_O_3_ or FeSO_4_ acts as an electron donor under aerobic conditions (O_2_ as an electron acceptor). The cells are regular to irregular cocci with a diameter of 0.8–1.5 μm (Additional file 1: Figure S2).

#### Genomic DNA preparation, genome sequencing and assembly

HS-1^T^ was cultivated in a 5 L glass bottle using ~ 4 L modified Brock’s basal salt (MBS) medium [7], supplemented with yeast extract (1 g/L) and glucose (1 g/L) under aerobic conditions (65 °C, pH 3). Approximately 1 L of the culture in early exponential phase was centrifuged (OD_600_ = ~ 0.1, 8000×*g*, 4 °C, 10 min), and the supernatant was removed. DNA was extracted from the resulting cell pellets using the Genomic DNA Buffer Set (QIAGEN) and the Genomic-Tip 500/G (QIAGEN), according to the manufacturer’s protocols. The quantity and purity of the extracted DNA was checked spectrophotometrically and through agarose gel electrophoresis. The genome sequencing of the *S. acidiphilus* strain HS-1^T^ was performed by Macrogen Inc. (South Korea) using a PacBio RS II sequencer (Pacific Biosciences, Menlo Park, CA, US). De novo assembly was conducted using the Hierarchical Genome Assembly Process v.3.0 (https://github.com/PacificBiosciences/Bioinformatics-Training/wiki/HGAP).

#### Genome annotation

Annotation of the protein coding genes and the COG (cluster of orthologous groups) assignments were performed using the on-line annotation server DFAST [8]. The tRNA and rRNA genes were identified using tRNAscan-SE [9, 10] and DFAST, respectively. Pseudogenes were identified using LAST [11] implemented in DFAST. Protein coding genes with Pfam domains [12] were searched using a CD-search program [13] with an e-value threshold of less than 1*e*−2 (database: Pfam v.30.0). Signal peptides were predicted using PRED-SIGNAL [14]. Transmembrane helices were predicted using TMHMM [15]. CRISPR repeats were detected using the CRISPR recognition tool CRT [16]. Genes in internal clusters were predicted using the CD-HIT Suite (sequence identity cut-off: 0.3, minimal alignment coverage for longer and shorter sequences: 0.7, other parameters: default) [17].

#### Search of replication origin

The replication origin (*oriC*) and origin recognition box (ORB) in the chromosome were predicted by Ori-finder 2 [18]. Another replication origin in the chromosome was manually searched. Repeat sequences in the predicted *oriC* region were searched by the REPuter program [19].

#### Reexamination of chemolithotrophic growth on hydrogen

The autotrophic growth of HS-1^T^ on hydrogen was reexamined. MBS medium (10 mL, pH 3) was added to a glass test tube and the headspace was filled with H_2_/CO_2_/air (80:20:10, 120 kPa). A 50 μL of active culture was inoculated into the test tube and incubation occurred at 65 °C for 2 weeks.

#### Genome project history

The complete genome sequence of HS-1^T^ (= JCM 31740^T^ = InaCC Ar79^T^) was deposited in GenBank under accession number AP018553. The raw data (PacBio reads) used for the assembly was deposited in the DNA Data Bank of Japan under accession number DRA008516. The Bioproject accession number is PRJDB6753. A summary of the genome project is provided in Additional file 1: Table S2.

### Results and discussion

#### Genome properties

A complete circular genome sequence (2,353,189 bp) was successfully obtained from a total of 225,345 subreads (a total of 1,538,043,255 bp), and a plasmid was absent. The G+C content is 51.15 mol%, which is identical to the reported value of 52.0 mol% estimated by HPLC method [1]. The genome was predicted to contain a total of 2459 genes, of which 2411 code for proteins and 48 code for RNAs (rRNA: 3, tRNA: 45). The genome harbors each one copy of 5S, 16S and 23S rRNA genes. Genes of 16S and 23S rRNA are encoded in a gene cluster with a 191 bp spacer region, while 5S rRNA gene is found in different location. Among the 2411 protein coding genes, 1219 were assigned putative functions. A total of 837 and 244 genes could be assigned COG functional categories and pseudogenes, respectively (Additional file 1: Tables S3, S4, Figure S3). Three CRISPR repeat regions were detected (positions: 1664810–1679609, 1679646–1680862, and 1688612–1701897). No plasmids were detected. Other genomic statistics such as predicted Pfam domains, signal peptides, and transmembrane helices are summarized in Additional file 1: Table S3.

#### Insights from the genome sequence

##### Replication initiation genes and *oriC*

**Fig. 1:**
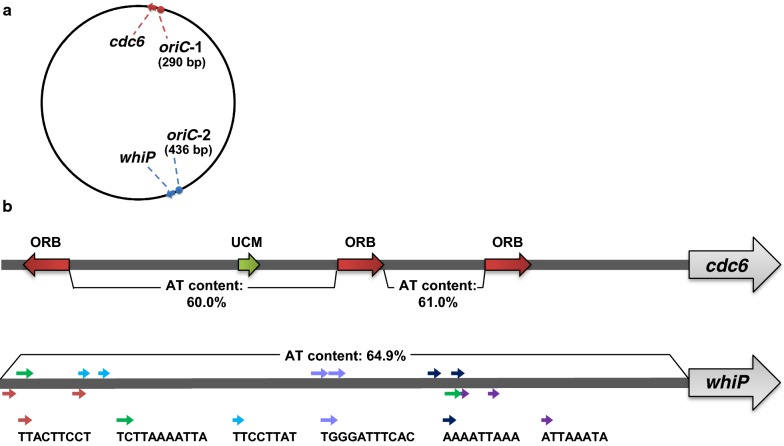
Predicted *oriCs* in the *Sulfodiicoccus acidiphilus* HS-1^T^ genome. **a** Positions of the predicted *oriC*s, **b** ORB, UCM and repeat sequences found upstream of *cdc6* and *whiP*

All previously identified species of the order *Sulfolobales* (whose complete genomic sequences are available) have three copies of the *cdc6* gene (a cell division control gene, Additional file 1: Table S5). In contrast, only one copy of the *cdc6* gene (HS1genome0091) was found in the HS-1^T^ genome (Fig. 1a). One replication origin (named *oriC*-1, position: 87,046–87,335) located directly upstream of the *cdc6* gene was predicted by the Ori-finder 2. The *oriC*-1 had a relatively high content of adenine and thymine residues (A/T rich) (56.55%) and contained three ORBs as typical archaeal genomes with the following sequence: ACCCCTCTGTTTCCACTGGA [18, 20–22] (Fig. 1b).A previously reported uncharacterized motif (UCM) that exists in the *oriC* regions of several *Crenarchaea* [23] has also been located in HS-1^T^ *oriC*-1. These facts indicate that *oriC*-1 is a DNA replication origin. Another replication initiation-related gene, *whiP* (HS1genome1070) [23], was found on the opposite side of the *cdc6* (Fig. 1a), however, neither oriC nor ORB sequences were found around *whiP* by Ori-finder 2. We then manually searched for putative *oriC* regions, and an A/T rich intergenic region (64.91%, position: 1,016,172–1,016,607, named as *oriC*-2) was found directly upstream of *whiP*. Further attempts to search for repeat sequences in *oriC*-2 using the REPuter program [19] confirmed the presence of 12 direct repeats (8–11 bp, Fig. 1b). The role of the direct repeats is unclear, although they may be involved in the recognition of the replication origin for the DNA initiation protein WhiP. Perhaps the direct repeats alternatively function as an ORB. Further experiments such as DNaseI footprint analyses [23, 24] are required to know the function.

#### Pathways involved in autotrophic growth

**Fig. 2:**
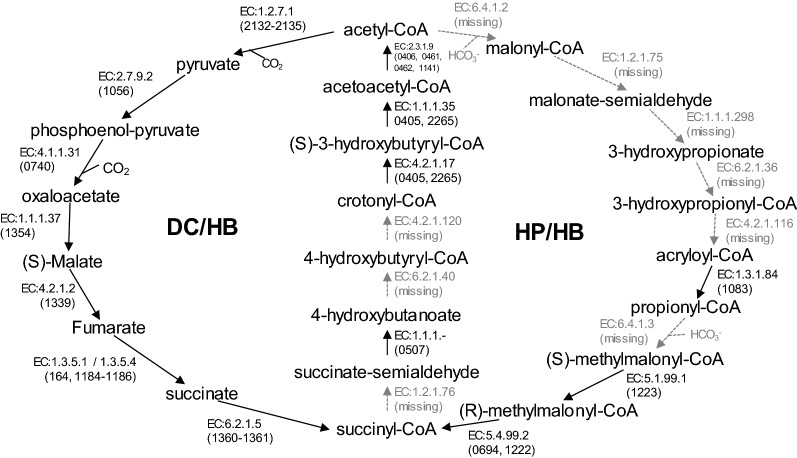
Incomplete DC/HB and HP/HB cycles of *Sulfodiicoccus acidiphilus* HS-1^T^. The annotated ORF number is shown in parentheses. Gray arrows indicate pathways that are missing in HS-1^T^. HP/HB: 3-hydroxypropionate/4-hydroxybutyrate cycle; DC/HB: dicarboxylate/4-hydroxybutyrate cycle

Two CO_2_ fixation pathways have been reported in the thermophilic autotroph *Crenarchaeota*, namely the 3-hydroxypropionate/4-hydroxybutyrate (HP/HB) cycle and the dicarboxylate/4-hydroxybutyrate (DC/HB) cycle [25]. Most species in the order *Sulfolobales* are thought to possess the HP/HB cycle but not the DC/HB cycle [25–27]. Both cycles are absent in HS-1^T^ (Fig. 2). The other known autotrophic pathways (Calvin–Bassham–Benson cycle, reductive citric acid cycle (Amon–Buchanan cycle), reductive acetyl-CoA pathway (Wood–Ljungdahl pathway) and 3-hydroxypropionate cycle) [28] have not been identified in HS-1^T^. These observations are consistent with the incapability of chemolithoautotrophic growth of HS-1^T^ [1]. In the previous paper, we mentioned the capability of chemolithoautotrophic growth of HS-1^T^ using hydrogen as an electron donor. Based on the genomic information, we carefully reexamined the capability of chemolithoautotrophic growth with serial inoculation using the chemolithoautotrophic medium, and no growth occurred after 2nd inoculation. Thus, we revise our previous description regarding the autotrophic growth of HS-1^T^ on hydrogen, to “HS-1^T^ does not grow on hydrogen autotrophically.”

##### Lack of *polB3* in the genome of HS-1^T^

**Fig. 3:**
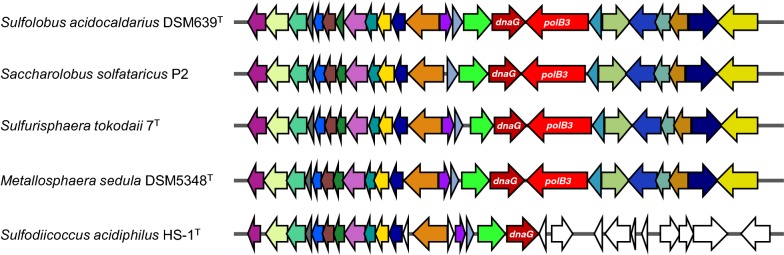
Organization of genes around *dnaG* and *polB3* in the genomes of the *Sulfolobales* species. The downstream *dnaG* region, containing *polB3* and several other genes, is not present in the HS-1^T^ genome. Each color represents similar genes except for white, which indicates genes detected only in this region of HS-1^T^

Three groups of family B DNA polymerases (PolB1, PolB2, and PolB3) have been associated with archaea: PolB1 is only distributed in the superphylum that includes *Thaumarchaeota*, *Aigarchaeota*, *Crenarchaeota* and *Korarchaeota* (also known as TACK superphylum); PolB2 is patchily distributed in most of the archaeal lineages; and PolB3 is distributed in all archaea except for *Thaumarchaeota* [29]. Surprisingly, the HS-1^T^ genome lacks *polB3*, although all the crenarchaeal genomes reported before harbor the gene. In the order *Sulfolobales*, *polB3* is located on the downstream region of *dnaG*, with a high genomic synteny around the *dnaG* sequence among *Sulfolobales* species, while the genome structure of the downstream region of dnaG of HS-1^T^ is different from those in the genomes of other *Sulfolobales* species (Fig. 3). Since all the other species in the order *Sulfolobales* have a *polB3* downstream of a *dnaG*, HS-1^T^ may have lost *polB3* and the downstream region of *dnaG* during the course of its evolution. The roles of PolB1, PolB2 and PolB3 in *Saccharolobus solfataricus* (synonym: *Sulfolobus solfataricus*), a model organism of the order *Sulfolobales*, were previously investigated in vivo by Choi et al. [30]. The authors showed that PolB1 was catalytically much more efficient and processive than PolB2 and PolB3, suggesting that PolB1 plays a catalytic role as the main replicative DNA polymerase. They also suggested that PolB2 and PolB3 have limited catalytic roles in translesion DNA synthesis and may not be involved in chromosomal DNA replication [30]. The lack of a *polB3* gene in *S. acidiphilus* HS-1^T^ indicates that PolB3 is not essential for either DNA replication or translesion DNA synthesis in *Sulfolobales* or the phylum *Crenarchaeota*.

## Limitations

This study focused on noteworthy genomic features of *S. acidiphilus* HS-1^T^ that are distinct from other *Sulfolobales* species. Although our genomic analyses revealed some exceptions of genomic features in *Sulfolobales*, further molecular biology assessment and biochemical analyses are needed to resolve the issues raised in this manuscript.

## Additional file

**Additional file 1: Table S1.** Classification and general features of *Sulfodiicoccus acidiphilus* strain HS-1^T^. **Table S2.** Project information. **Table S3.** Genome statistics. **Table S4.** Number of genes associated with general COG functional categories. **Table S5.** Number of *cdc6* and *whiP* genes in the order *Sulfolobales*. **Figure S1.** The maximum likelihood phylogenetic tree of the order *Sulfolobales* based on the 16S rRNA gene. **Figure S2.** Scanning electron micrographs of *Sulfodiicoccus acidiphilus* HS-1^T^. **Figure S3.** A circular map of the *S. acidiphilus* strain HS-1^T^ genome.

## Data Availability

The complete genome sequence of HS-1^T^ is available in GenBank repository, https://www.ncbi.nlm.nih.gov/genbank/, under accession number AP018553 (the Bioproject accession number is PRJDB6753). The raw data (PacBio reads) used for the assembly is available in the DNA Data Bank of Japan repository, https://www.ddbj.nig.ac.jp/, under accession number DRA008516.

## Abbreviations

HPLC: high performance liquid chromatography
HP/HB: 3-hydroxypropionate/4-hydroxybutyrate
DC/HB: dicarboxylate/4-hydroxybutyrate
ORB: origin recognition box
UCM: uncharacterized motif
