# Supplementary material for: Complete genome sequence of the Sulfodiicoccus acidiphilus strain HS-1T, the first crenarchaeon that lacks polB3, isolated from an acidic hot spring in Ohwaku-dani, Hakone, Japan
Source: BMC Res Notes. 2019 Jul 22;12:444. doi: 10.1186/s13104-019-4488-5 (PMC6647314; doi:10.1186/s13104-019-4488-5)
Supplement: Supplementary file 1 — Additional file 1: Table S1. Classification and general features of Sulfodiicoccus acidiphilus strain HS-1T. Table S2. Project information. Table S3. Genome statistics. Table S4. Number of genes associated with general COG functional categories. Table S5. Number of cdc6 and whiP genes in the order Sulfolobales. Figure S1. The maximum likelihood phylogenetic tree of the order Sulfolobales based on the 16S rRNA gene. Figure S2. Scanning electron micrographs of Sulfodiicoccus acidiphilus HS-1T. Figure S3. A circular map of the S. acidiphilus strain HS-1T genome. [file 13104_2019_4488_MOESM1_ESM.pdf]

Table S1. Classification and general features of *Sulfodiicoccus acidiphilus* strain HS-1^T [1]

MIGS ID[a]	Property	Term	Evidence code[b]
	Classification	Domain *Archaea*	TAS [2]
		Phylum *Crenarchaeota*	TAS [3]
		Class *Thermoprotei*	TAS [4]
		Order *Sulfolobales*	TAS [5]
		Family *Sulfolobaceae*	TAS [5]
		Genus *Sulfodiicoccus*	TAS [1]
		Species *Sulfodiicoccus acidiphilus*	TAS [1]
		Type strain: HS-1^T (Accession #s)	TAS [1]
	Gram stain	Not reported	TAS [1]
	Cell shape	Irregular cocci	TAS [1]
	Motility	Non-motile	TAS [1]
	Sporulation	Non-sporulating	NAS
	Temperature range	50–70 °C	TAS [1]
	Optimum temperature	65–70 °C	TAS [1]
	pH range; Optimum	1.4–5.5; 3.0–3.5	TAS [1]
	Carbon source	yeast extract, beef extract, casamino acids, peptone, tryptone, xylose, galactose, glucose, maltose, sucrose, raffinose, lactose, aspartic acid, glutamic acid	TAS [1]
MIGS-6	Habitat	Acidic terrestrial hot spring	TAS [1]
MIGS-6.3	Salinity	0–2.5% (w/v) NaCl	TAS [1]
MIGS-22	Oxygen requirement	Aerobic	TAS [1]
MIGS-15	Biotic relationship	Free-living	TAS [1]
MIGS-14	Pathogenicity	Non-pathogenetic	NAS
MIGS-4	Geographic location	Hakone, Ohwaku-dani, Kanagawa, Japan	TAS [1]
MIGS-5	Sample collection	June 2014	TAS [1]
MIGS-4.1	Latitude	35.239	TAS [1]
MIGS-4.2	Longitude	139.019	TAS [1]
MIGS-4.4	Altitude	Surface	TAS [1]

[a] MIGS: Minimum Information about the Genome Sequence [6].

[b] Evidence codes - IDA: Inferred from Direct Assay; TAS: Traceable Author Statement (i.e., a direct report exists in the literature); NAS: Non-traceable Author Statement (i.e., not directly observed for the living, isolated sample, but based on a generally accepted property for the species, or anecdotal evidence). These evidence codes are from the Gene Ontology project [7].

Table S2. Project information.

MIGS[a] ID	Property	Term
MIGS 31	Finishing quality	Finished
MIGS-28	Libraries used	SMRTbell library
MIGS 29	Sequencing platforms	PacBio RS II sequencer
MIGS 31.2	Fold coverage	653.6×
MIGS 30	Assemblers	HGAP v. 3
MIGS 32	Gene calling method	DFAST
	Locus Tag	HS1genome
	Genbank ID	AP018553
	GenBank Date of Release	01-AUG-2018
	GOLD ID	Gp0324073
	BIOPROJECT	PRJDB6753
MIGS 13	Source Material Identifier	HS-1^T, JCM 31740^T, InaCC Ar79^T
	Project relevance	Evolution

[a] MIGS: Minimum Information about the Genome Sequence [6].

Table S3. Genome statistics.

Attribute	Value	% of Total
Genome size (bp)	2,353,189	n/a
DNA coding (bp)	1,751,769	74.44
DNA G+C (bp)	1,203,539	51.15
DNA scaffolds	1	n/a
Total genes	2459	100.00
Protein coding genes	2411	98.05
RNA genes	48	1.95
Pseudo genes	244	9.92
Genes in internal clusters	163	6.63
Genes with function prediction	1267	51.53
Genes assigned to COGs	837	34.04
Genes with Pfam domains	1713	69.66
Genes with signal peptides	161	6.54
Genes with transmembrane helices	442	17.97
CRISPR repeats	3	n/a

Table S4. Number of genes associated with general COG functional categories.

Code	Value	%age	Description
J	102	4.2	Translation, ribosomal structure and biogenesis
A	0	0.0	RNA processing and modification
K	56	2.3	Transcription
L	28	1.2	Replication, recombination and repair
B	2	0.1	Chromatin structure and dynamics
D	9	0.4	Cell cycle control, Cell division, chromosome partitioning
V	23	1.0	Defense mechanisms
T	17	0.7	Signal transduction mechanisms
M	42	1.7	Cell wall/membrane biogenesis
N	9	0.4	Cell motility
U	8	0.3	Intracellular trafficking and secretion
O	32	1.3	Posttranslational modification, protein turnover, chaperones
C	83	3.4	Energy production and conversion
G	73	3.0	Carbohydrate transport and metabolism
E	104	4.3	Amino acid transport and metabolism
F	34	1.4	Nucleotide transport and metabolism
H	58	2.4	Coenzyme transport and metabolism
I	57	2.4	Lipid transport and metabolism
P	43	1.8	Inorganic ion transport and metabolism
Q	24	1.0	Secondary metabolites biosynthesis, transport and catabolism
R	124	5.1	General function prediction only
S	16	0.7	Function unknown
-	1574	65.3	Not in COGs

The total is based on the total number of protein coding genes in the genome.

Table S5. Number of *cdc6* and *whiP* genes in the order *Sulfolobales* [a].

Species	No. of *cdc6*	No. of *whiP*
Sulfodiicoccus acidiphilus HS-1^{T} (AP018553)	1	1
Sulfolobus acidocaldarius DSM 639^{T} (GCF_000012285)	3	1
Saccharolobus solfataricus P2 (GCF_000007005)	3	1
Sac. solfataricus P1^{T} (GCA_900079115)	3	1
Sulfurisphaera tokodaii 7^{T} (GCF_000011205)	3	1
Metallosphaera sedula TH-2^{T} (GCA_000016605)	3	1
M. cuprina Ar-4^{T} (GCF_000204925)	3	1
'*Acidianus hospitalis*' W1 (GCA_000213215)	3	1
'*A. manzaensis*' YN-25 (GCA_002116695)	3	1
'*A. copahuensis*' ALE1 (GCA_000632495)	3	1
Sulfolobus sp. JCM 16833 (GCA_001316085)	3	1

[a] The numbers were predicted from annotated genes in each of complete or draft genome sequences those that are available in the GenBank DNA database except for *S. acidocaldarius* and *S. solfataricus*, whose *cdc6* genes were experimentally examined previously. Genbank accession numbers are indicated in the parentheses.

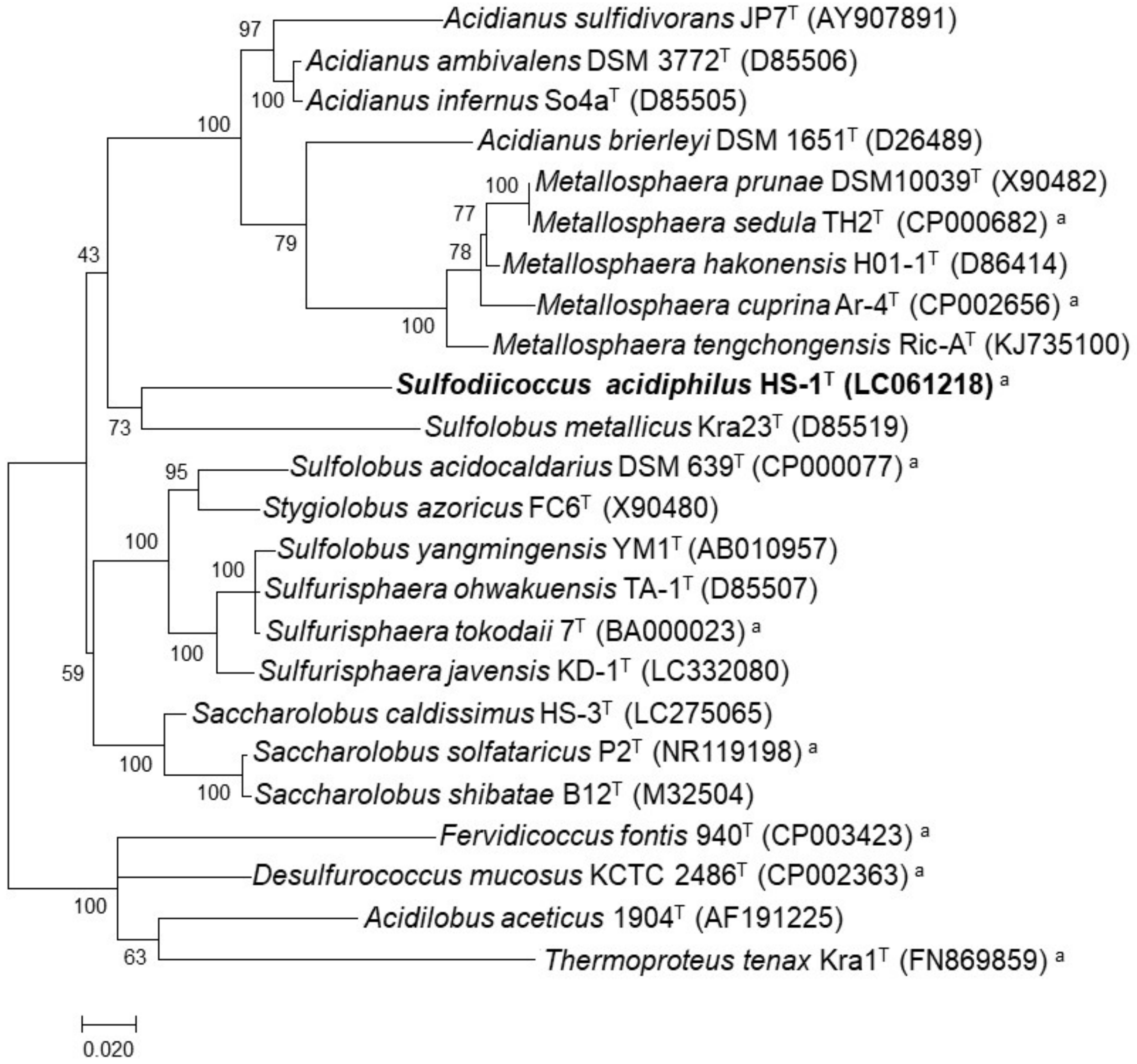

Figure S1. The maximum likelihood phylogenetic tree of the order *Sulfolobales* based on the 16S rRNA gene. The bootstrap values (1000 sampling) higher than 50% are shown at nodes. *Fervidicoccus fontis* 940^T, *Desulfurococcus mucosus* KCTC 2486^T, *Acidilobus aceticus* 1904^T, and *Thermoproteus tenax* Kra1^T were used as an out group. Sequence accession numbers are indicated in parentheses. The bar indicates the number of nucleotide substitutions per position. [a] Species whose complete genome sequence is available.

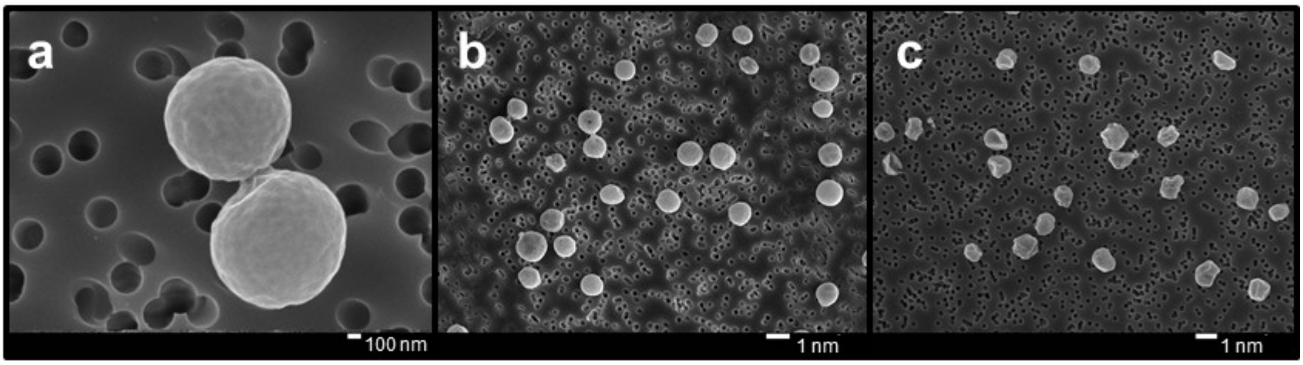

Figure S2. Scanning electron micrographs of *Sulfodiicoccus acidiphilus* HS-1^{T} in the exponential phase (a–b) and the stationary phase (c).

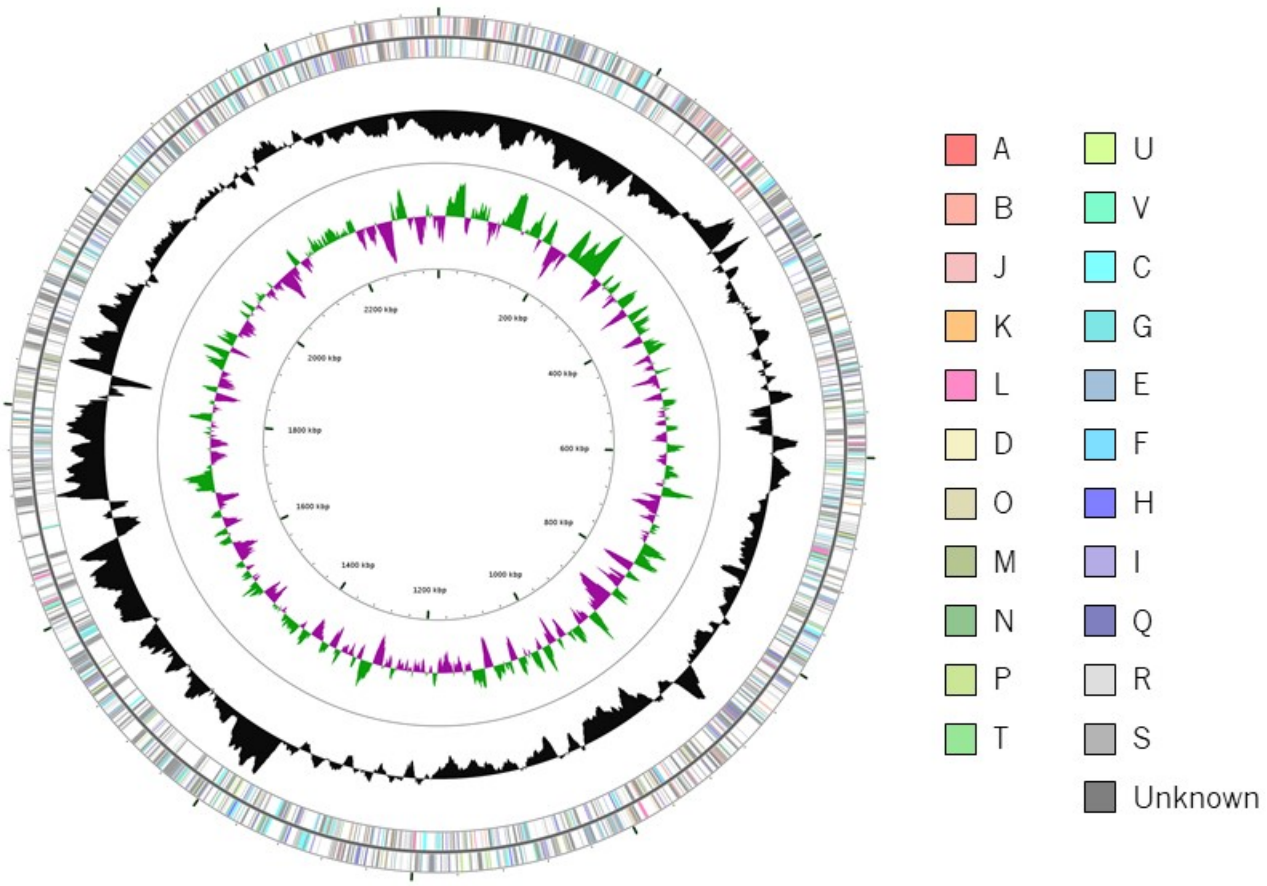

Figure S3. A circular map of the *S. acidiphilus* strain HS-1^T genome. From outside to center: genes on the forward strand (colored by COG categories), genes on the reverse strand (colored by COG categories), G + C content (black), and G + C skew (green and pink).

References for additional files

1. Sakai HD, Kurosawa N. *Sulfodiicoccus acidiphilus* gen. nov., sp. nov., a sulfur-inhibited thermoacidophilic archaeon belonging to the order *Sulfolobales* isolated from a terrestrial acidic hot spring. Int J Syst Evol Microbiol. 2017;67:1880–6.

2. Woese CR, Kandler O, Wheelis ML. Towards a natural system of organisms: proposal for the domains Archaea, Bacteria, and Eucarya. Proc Natl Acad Sci U S A. 1990;87:4576–9.

3. Garrity GM, Holt JG. The Road Map to the Manual. In: Garrity G, Boone DR, Castenholz RW, editors. Bergey's Man Syst Bacteriol. Second edi. New York: Springer; 2001. p. 119–69.

4. Reysenbach AL. Class I. *Thermoprotei* class. nov. 2nd ed. Garrity GM., Boone DR., Castenholz RW., editors. Bergey's Man. Syst. Bacteriol. New York: Springer; 2001.

5. Stetter KO. Order III. *Sulfolobales* ord. nov. Family *Sulfolobaceae* fam. nov. In: Staley JT, Bryant MP, Pfennig N, Holt J, editors. Bergey's Man Syst Bacteriol. First Edit. Baltimore: The Williams and Wilkins Co; 1989. p. 2250–1.

6. Field D, Garrity G, Gray T, Morrison N, Selengut J, Sterk P, et al. The minimum information about a genome sequences (MIGS) specification. Nat Biotechnol. 2008;26:541–7.

7. Ashburner M, Ball CA, Blake JA, Botstein D, Butler H, Cherry JM, et al. Gene ontology: Tool for the unification of biology. Nat Genet. 2000;25:25–9.
